# Isolation, Structural Analyses and Biological Activity Assays against Chronic Lymphocytic Leukemia of Two Novel Cytochalasins — Sclerotionigrin A and B

**DOI:** 10.3390/molecules19079786

**Published:** 2014-07-08

**Authors:** Lene M. Petersen, Tanja T. Bladt, Claudia Dürr, Martina Seiffert, Jens C. Frisvad, Charlotte H. Gotfredsen, Thomas O. Larsen

**Affiliations:** 1 Department of Systems Biology, Technical University of Denmark, Søltofts Plads B221, DK-2800 Kgs. Lyngby, Denmark; E-Mails: lmape@bio.dtu.dk (L.M.P.); ttb@bio.dtu.dk (T.T.B.); jcf@bio.dtu.dk (J.C.F.); 2 German Cancer Research Center, Molecular Genetics, Im Neuenheimer Feld 280, D-69120 Heidelberg, Germany; E-Mails: C.Duerr@dkfz-heidelberg.de (C.D.); M.Seiffert@dkfz-heidelberg.de (M.S.); 3 Department of Chemistry, Technical University of Denmark, Kemitorvet, B201, DK-2800 Kgs. Lyngby, Denmark; E-Mail: chg@kemi.dtu.dk

**Keywords:** cytochalasins, apergilli, *Apsergillus sclerotioniger*, chronic lymphocytic leukemia

## Abstract

Two new cytochalasins, sclerotionigrin A (**1**) and B (**2**) were isolated together with the known proxiphomin (**3**) from the filamentous fungus *Aspergillus sclerotioniger*. The structures and relative stereochemistry of **1** and **2** were determined based on comparison with **3**, and from extensive 1D and 2D NMR spectroscopic analysis, supported by high resolution mass spectrometry (HRMS). Compounds **2** and **3** displayed cytotoxic activity towards chronic lymphocytic leukemia cells *in vitro*, with **3** being the most active.

## 1. Introduction

Chronic lymphocytic leukemia (CLL) is the most common type of leukemia among adults in the Western World. CLL is considered an incurable disease and today’s applied treatment strategies primarily aim at prolonging patient survival [1,2]. Consequently discovery of compounds that act against CLL and other types of cancer cells is crucial. Numerous types of anticancer compounds have been reported in the literature [3,4], and with the increase in specific biological assays, both novel and previously described compounds might display promising novel bioactivities [5,6]. An important and diverse group of fungal anticancer compounds that have caught our interest due to their wide range of biological functions are the cytochalasans [7]. In particular this includes inhibitory activities towards lung, ovarian, and human colon cancer as well as human leukemia [8,9]. Recently, we have demonstrated that chaetoglobosin A, produced by *Penicillium aquamarinum*, selectively induces apoptosis in CLL cells with a median lethal concentration (LC_50_) value of 2.8 µM [10]. Encouraged by this finding we searched for potential novel cytochalasan type of compounds in black aspergilli.

The only documented indication of production of cytochalasans in *Aspergillus* subgenus *Circumdati* section *Nigri* is aspergillin PZ, for which aspochalasin C or D has been suggested as precursor [11,12]. However in the sister clade *Aspergillus* subgenus *Circumdati* section *Flavipedes*, numerous cytochalasans have been reported [13,14,15,16,17,18,19,20,21,22,23,24,25,26] including aspochalasin A–D [27,28]. Also in another species of *Aspergillus* subgenus *Circumdati* section *Circumdati*, *Aspergillus elegans* several cytochalasins were found, including aspergillin PZ [29], supporting the view that aspochalasin D is a precursor of aspergillin PZ. Finally in the less closely related *Aspergillus clavatus* in *Aspergillus* subgenus *Fumigati* section *Clavati*, cytochalasin E and K have been isolated [30,31].

Here we report the target-guided isolation and structure elucidation based on UV, MS, and NMR data of the two novel cytochalasins sclerotionigrin A (**1**) and B (**2**). Compounds **1** and **2** were isolated from *Aspergillus sclerotioniger* (IBT 22905) together with the known cytochalasin proxiphomin (**3**) [32]. We have previously reported ochratoxin A, ochratoxin B and pyranonigrin A from this isolate [33].

## 2. Results and Discussion

**Figure 1:**
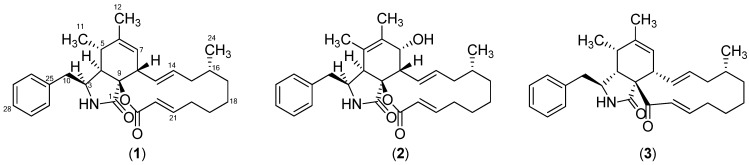
Structures of sclerotionigrin A (**1**), sclerotionigrin B (**2**) and proxiphomin (**3**).

The structure of compound **3** (Figure 1) was tentatively identified through UHPLC-DAD-HRMS based dereplication of the crude extract. The pseudomolecular ion, [M+H]^+^, was recognized from the mass spectrum due to the presence of the sodiated adduct, [M+Na]^+^ and the corresponding dimeric adducts [2M+H]^+^ and [2M+Na]^+^. The molecular formula C_29_H_37_NO_2_ was established with an accuracy of 0.8 ppm through the monoisotopic mass of [M+H]^+^ of *m/z* 432.2901. The formula was used for a query in Antibase2012 [34] with one resulting hit, proxiphomin (**3**). NMR data and optical rotation of **3** matched published data [32].

Compound **1** was purified as a yellow powder. The UV spectrum displayed an absorption maximum at 210 nm. The ESI^+^ spectrum showed a distinct adduct pattern consisting of [M+H]^+^, [M+Na]^+^, [2M+H]^+^ and [2M+Na]^+^. The molecular formula C_29_H_37_NO_3_ (12 double-bond equivalents) was obtained from HRMS of [M+H]^+^ (*m/z* 448.2843) with an accuracy of 2.3 ppm. The ^1^H-NMR spectrum revealed the presence of one amide proton, 15 methines (five which were vinylic and five aromatic), six methylenes, and three methyls (Table 1).

**Table 1:** NMR data for sclerotionigrin A (**1**) ^†^.

No.	δ_H_ (Integral, Mult., *J* [Hz])	δ_C_	HMBC	NOESY
1	-	170.6	-	-
2	8.00 (1H, s)	-	1, 3, 4, 9	3, 10
3	3.09 (1H, td, 5.8, 3.1)	54.2	-	2, 4, 10, 11, 12, 26/30
4	2.53 (1H, dd, 4.2, 3.1)	49.1	3, 5, 6, 9	3, 10, 11, 26/30
5	2.57 (1H, m)	33.6	-	7, 8, 11
6	-	140.0	-	-
7	5.25 (1H, m)	123.6	-	5, 8, 12
8	3.15 (1H, m)	45.8	-	5, 7, 13, 14
9	-	85.4	-	-
10	2.82 (2H, m)	42.6	3, 4, 25, 26/30	3, 4, 26/30
11	0.68 (3H, d, 7.1)	12.8	4, 5, 6	3, 4, 5, 12, 26/30
12	1.65 (3H, s)	19.4	5, 6, 7	3, 7, 11
13	5.85 (1H, ddd, 14.8, 10.0, 1.2)	128.9	15	8, 15
14	5.22 (1H, m)	132.6	8	8, 15', 16
15	1.60 (1H, d, 13.5)	40.7	13, 14, 16	13, 15'
15'	2.07 (1H, dd, 13.5, 2.1)	40.7	-	14, 15, 16, 20', 24
16	1.36 (1H, m)	31.9	-	14, 15', 19'
17	0.61 (1H, m)	33.8	-	17', 18', 24
17'	1.66 (1H, m)	33.8	-	17
18	1.14 (1H, m)	25.9	-	18'
18'	1.53 (1H, m)	25.9	-	17, 18
19	1.29 (1H, m)	25.3	-	24
19'	1.68 (1H, m)	25.3	-	16, 21
20	2.23 (1H, m)	33.1	-	20', 21
20'	2.29 (1H, m)	33.1	-	15', 20, 22
21	6.96 (1H, ddd, 15.5, 8.6, 6.8)	151.7	23	19', 20, 22
22	5.65 (1H, d, 15.5)	120.6	20, 23	20', 21
23	-	163.5	-	-
24	0.84 (3H, d, 6.3)	20.0	15, 16, 17	15', 17, 19
25	-	137.8	-	-
26 ^‡^	7.14 (1H, app. d, 7.5)	129.5	10, 26/30, 28	3, 4, 10, 11
27 ^‡^	7.26 (1H, app. t, 7.4)	128.0	25, 29	-
28	7.18 (1H, app. t, 7.5)	126.1	26, 30	-
29 ^‡^	7.26 (1H, app. t, 7.5)	128.0	25, 27	-
30 ^‡^	7.14 (1H, app. d, 7.5)	129.5	10, 26/30, 28	3, 4, 10, 11

^†^^1^H NMR data were obtained at 500 MHz in DMSO-*d_6_* and ^13^C data were obtained at 125 MHz in DMSO-*d_6_*. ^13^C-NMR chemical shifts were determined from HSQC and HMBC experiments; ^‡^ It was not possible to distinguish between No. 26 and 30 as well as No. 27 and 29.

**Figure 2:**
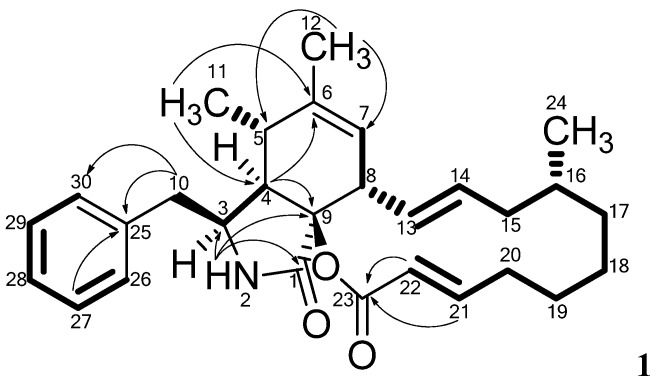
Important HMBC correlations connecting the four COSY spin systems (marked in bold) in **1**. The remaining HMBC correlations are found in Table 1.

The DQF-COSY spectrum of **1** defined four spin systems. The linking between COSY spin systems and assignments of the remaining signals and quaternary carbons were accomplished through detailed analysis of HMBC experimental data (Figure 2).

The HMBC correlations from the protons at δ_H_ 2.82 ppm (H10) and 7.26 (H27 and H29) to a quaternary carbon at δ_C_ 137.8 (C25), together with HMBC correlations from the protons at δ_H_ 7.14 (H26 and H30) to the carbon at δ_C_ 42.6 (C10) linked two of the spin systems belonging to the Phe moiety in **1**. The amide proton at δ_H_ 8.00 ppm (H2) displayed HMBC correlations to the carbons at δ_C_ 54.2 (C3) and 170.6 ppm (C1). Combination of these HMBC correlations established the Phe moiety of **1**, which was incorporated on the polyketide (PK) part of the molecule.

The PK part of **1** could be established through a large COSY spin system (from H7 to H22), equal to that seen in **3**. Furthermore a COSY coupling was found between the proton at δ_H_ 2.57 ppm (H5) and a methyl group at 0.68 ppm (H11). This part was coupled to the PK part by a weak COSY coupling between H5 and H7 identified as a ^4^*J* alyllic coupling. The COSY spin system could furthermore be connected via HMBC correlations to the above mentioned Phe moiety as well as the PK part. The protons at δ_H_ 1.65 (H12) correlated to the carbons at δ_C_ 33.6 (C5) and 123.6 ppm (C7) where the proton at δ_H_ 0.68 (H11) correlated to the carbons at δ_C_ 49.1 (C4) and 140.0 ppm (C6). The proton at 2.53 ppm (H4) correlated to C5, C6 and the quaternary carbon at δ_C_ 85.4 ppm (C9). Finally the PK chain was closed via an ester bond assigned from HMBC correlations from the vinylic protons at δ_H_ 5.65 (H22) and 6.96 ppm (H21) to the carbonyl carbon at δ_C_ 163.5 ppm (C23) supported by the high chemical shift of the quaternary carbon at δ_C_ 85.4 ppm (C9) indicating that C9 is bound to oxygen, and a carbonyl group, similar to what is seen in several other cytochalasans [7].

This structure accounted for all the degree of unsaturation required by the formula allowing the assignment of **1** as sclerotionigrin A. The size of the vicinal coupling constants (^3^*J*_HH_) for H13/H14 and H21/H22 were rather large (14.8 and 15.5 Hz respectively) suggesting a *trans* stereochemistry. NOESY experiments enabled determination of the relative stereochemistry for most of the stereogenic centers of **1**. NOE connectivities were found between the proton at δ_H_ 3.09 ppm (H3), δ_H_ 2.53 (H4) and the methyl at δ_H_ 0.68 ppm (H11) placing these protons at the same side of the central ring system, which to the best of our knowledge has not been reported for other cytachalasins. Other NOE connectivities were observed between the protons at δ_H_ 2.57 ppm (H5), 5.25 (H7) and 3.15 (H8), whereas no NOE connectivities could be seen from either of these to H3, H4 or H11, strongly indicating the positioning of H5, H7 and H8 on the opposite side of the central ring system compared to H3, H4 and H11. The stereocenters at C9 and C16 could not be assigned through NOESY connectivities; however being biosynthesized by the same fungus we propose that the stereochemistries at these centers are identical to those of **3**. Especially we note that the extra oxidation between C9 and C23 in other cytochalasans never leads to a change in stereochemistry at C9 [7,15]. We do however note that the optical rotation of **1** and **2** are positive as opposed to that of **3** and other similar cytochalasans [15], indicating a possible difference in stereochemistry, which could be accounted for by the change of stereochemistry at C4. Further experiments, e.g., X-ray crystallography or circular dichroism (CD) are therefore needed to clarify the absolute stereochemistry of **1**.

Compound **2** was isolated as a yellow powder, and displayed a UV absorption maximum at 212 nm and the ESI^+^ MS adducts [M+H]^+^, [M+Na]^+^, [2M+H]^+^ and [2M+Na]^+^. The molecular formula of **2**, C_29_H_37_NO_4_ was deduced from the monoisotopic mass obtained from the [M+H]^+^ ion (*m/z* 464.2797) with an accuracy of 1.2 ppm. Examination of the NMR spectra of **2** displayed a high similarity compared to **1**. Comparison of the NMR spectra of **1** and **2** (see Table 1 and Table 2, respectively) revealed that the difference between them is located in positions five, six and seven. The Phe moiety in **2** was identified through a connection of the two COSY spin systems linked by HMBC correlations as demonstrated for **1**. The COSY spin system of the PK chain terminated with a proton at δ_H_ 3.68 ppm (H7), indicating a binding to a hydroxyl group instead of the vinylic methine group observed for **1** at this position. This was also evident from the carbon chemical shift moving to δ_C_ 69.1 ppm (C7).

**Figure 3:**
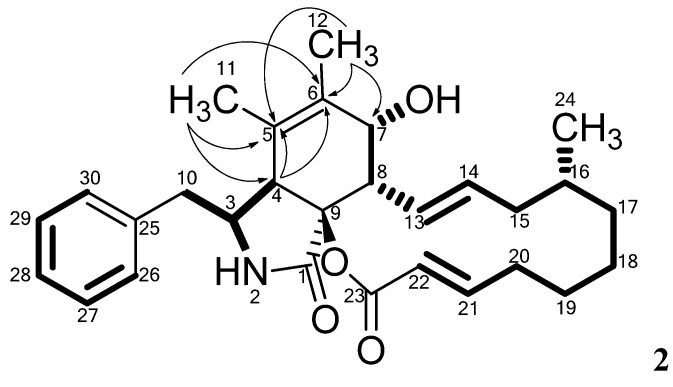
Important HMBC correlations establishing the quaternary carbon C5 and C6 in **2**. The remaining HMBC correlations are found in Table 2. Individual COSY spin systems are marked in bold.

HMBC correlations from the three protons of the methyl group at δ_H_ 1.52 ppm (H12) to the carbons at δ_C_ 123.9 (C5), 134.2 (C6) and 69.1 (C7), combined with correlations from the protons at δ_H_ 1.16 ppm (H11) to the carbons at δ_C_ 47.1 (C4) and 123.9 ppm (C5) and 134.2 (C6) linked the Phe moiety to the spin system in the polyketide chain (Figure 3). The remaining chemical shifts in **2** matched the chemical shifts of **1** (Table 1 and Table 2) and the structure of **2** was established altogether giving a classic methylated cytochalasin carbon skeleton [7].

The relative stereochemistry of **2** was established partly through NOE connectivities (Table 2) and shown to be very similar to that of **1**. Connectivities between H3 and H4 were however not confirmed in **2**, since these resonances were overlapping with the water resonance (Supplementary Figure S10). The optical rotation of **2** was positive like the optical rotation of **1**, also indicating that the two compounds have the same relative stereochemistry. The absolute stereochemistry of **2** has not yet been solved.

Biological testing of the cytotoxicity of compounds **1**–**3** towards CLL cells *in vitro* was performed using a CellTiter-Glo^®^ assay [10]. Compound **3** displayed the strongest effects, with estimated LC_50_ values of ca. 48 µM whereas no effect was found towards healthy B-cells in concentrations <100 µM. Compounds **1** showed minor activity at a concentration of 72 µM, while **2** did not have any effect (Table 3 and Supplementary Table S2, Figures S17, S18), indicating that the novel stereochemistry in the central ring system of **1** and **2** has a negative effect on target interactions. Due to the low anticancer activities of the sclerotionigrins, no further investigations proving their exact mode of action were undertaken.

**Table 2:** NMR data for sclerotionigrin B (2) ^ †^.

No.	δ_H_ (Integral, Mult., *J* [Hz])	δ_C_	HMBC	NOESY
1	-	171.2	-	-
2	8.34 (1H, br. s)	-	3, 4, 9	3, 10'
3	3.39 (1H, m)	57.7	1, 4, 5, 9	2, 10, 10', 11, 26/30
4	3.32 (1H, m)	47.1	1, 5, 6, 9	13, 26/30
5	-	123.9	-	-
6	-	134.2	-	-
7	3.68 (1H, d, 9.7)	69.1	-	8, 12, 13
8	3.05 (1H, t, 10.0)	48.3	1, 4, 7, 9, 13, 14	7, 13, 14
9	-	83.6	-	-
10	2.55 (1H, dd, 13.0, 10.1)	42.5	3, 4, 25, 26/30	3, 10', 26/30
10'	2.92 (1H, dd, 13.0, 5.0)	42.5	3, 4, 25, 26/30	2, 3, 10, 26/30
11	1.16 (3H, s)	16.7	4, 5, 6	3, 26/30
12	1.52 (3H, s)	14.3	5, 6, 7	7
13	6.03 (1H, dd, 15.0, 11.3)	128.4	8, 15/15’	4, 7, 8, 14, 15
14	5.00 (1H, ddd, 15.0, 10.8, 3.4)	132.7	8, 15/15’	8, 13, 15, 15'
15	1.58 (1H, dt, 13.0, 11.1)	41.6	16	13, 14, 15', 16, 17'
15'	2.00 (1H, m)	41.6	-	14, 15, 16, 24
16	1.13 (1H, m)	32.5	-	15, 15', 17', 18, 24
17	0.52 (1H, m)	34.5	-	17'
17'	1.67 (1H, m)	34.5	24	15, 16, 17, 18, 24
18	0.86 (1H, m)	26.1	-	16, 17', 18'
18'	1.68 (1H, m)	26.1	20	18, 19, 21
19	1.30 (1H, m)	25.4	-	18', 19'
19'	1.73 (1H, m)	25.4	-	19
20	2.11 (1H, m)	33.4	-	20', 22
20'	2.41 (1H, m)	33.4	-	20, 21
21	6.89 (1H, ddd, 15.7, 10.8, 5.0)	151.4	20, 23	18', 20', 22
22	5.79 (1H, d, 16.1)	121.3	20, 23	20, 21
23	-	163.8	-	-
24	0.83 (3H, d, 6.6)	19.9	15, 16, 17	15', 16, 17'
25	-	137.4	-	-
26 ^‡^	7.08 (1H, app. d, 7.1)	128.9	10, 28, 30	3, 4, 10, 10', 11, 27/29
27 ^‡^	7.31 (1H, app. t, 7.5)	128.2	25, 29	26/30, 28
28	7.23 (1H, app. t, 7.4)	126.3	26, 30	27/29
29 ^‡^	7.31(1H, app. t, 7.5)	128.2	25, 27	26/30, 28
30 ^‡^	7.08 (1H, app. d, 7.1)	128.9	10, 26, 28	3, 4, 10, 10',11, 27/29

^†^^1^H-NMR data were obtained at 500 MHz in DMSO-*d_6_* and ^13^C data were obtained at 125 MHz in DMSO-*d_6_*. ^13^C-NMR chemical shifts were determined from HSQC and HMBC experiments; ^‡^It was not possible to distinguish between no. 26 and 30 as well as no. 27 and 29. The hydroxyl group at C7 is not observed, presumable because it overlaps with the water resonance.

**Table 3:** Estimated LC_50_ values for compound **1**–**3**.

Compound	CLL	Healthy B-Cells
Sclerotionigrin A (**1**)	72 µM	No effect
Sclerotionigrin B (**2**)	No effect	No effect
Proxiphomin (**3**)	48 µM	No effect

## 3. Experimental Section

### 3.1. Fungal Growth and Extraction

*Aspergillus sclerotioniger* (IBT 22905 = CBS 115572) is from the IBT culture collection at Department of Systems Biology, Technical University of Denmark. *A*. *sclerotioniger* was inoculated as three point inoculations on Czapek yeast agar (CYA) on 100 plates at 25 °C for 7 days in the dark. CYA plates were prepared as described by Samson *et al.* [35]. The plates were harvested and extracted twice overnight with ethyl acetate (EtOAc) containing 1% formic acid (FA). The extracts were filtered and concentrated *in vacuo*. Work up: The combined extract was dissolved in methanol (MeOH)/milliQ-water (water purified and deionized by a Millipore system through 0.22 μm membrane filter) (9:1) and an equal amount of heptane was added followed by separation of phases. Additional milliQ-water was added to the MeOH/water phase until a ratio of 1:1 was reached, and metabolites were extracted with dichloromethane (DCM). The phases were then concentrated separately *in vacuo*. The DCM phase was used for further fractionation.

### 3.2. Preparative Isolation of Cytochalasins

The extract was dry-loaded on diol resin and fractionated on a 50 g pre-packed diol column using an Isolera One automated flash purification system (Biotage, Uppsala, Sweden). The compounds were eluted using a seven step gradient of heptane-DCM-EtOAc-MeOH with a flow rate of 40 mL/min. Fractions were collected automatically (1 column volume in each fraction). The Isolera fractions were subjected to further purification on a semi-preparative HPLC Waters 600 Controller with a 996 photodiode array detector (Waters, Milford, MA, USA) on a Luna II C_18_ column 250 × 10 mm, 5 μm (Phenomenex, Torrance, CA, USA). A flowrate of 5 mL/min was used and 50% acetonitrile (ACN) isocratic for 5 min, then to 100% in 15 min. 50 ppm trifluoroacetic acid (TFA) was added to ACN and milliQ-water. This yielded **1** (7.8 mg), **2** (2.1 mg), and **3** (1.3 mg).

Sclerotionigrin A (**1**) Yellow solid; [α]_589.3nm_: +4° (*c* 0.8, MeOH); UV (ACN) λ_max_: 210 nm; HRMS *m/z* 448.2843 ([M + H]^+^ calculated for C_29_H_38_NO_3_, *m/z* 448.2853; 2.3 ppm); ^13^C- and ^1^H-NMR: see Table 1.

Sclerotionigrin B (**2**) Yellow solid; [α]_589.3nm_: +41° (*c* 0.2, MeOH); UV (ACN) λ_max_: 212 nm; HRMS *m/z* 464.2797 ([M + M]^+^ calculated for C_29_H_38_NO_4_, *m/z* 463.2724; 1.2 ppm); ^13^C- and ^1^H-NMR: see Table 2.

Proxiphomin (**3**) Yellow solid; [α]_589.3nm_: −21° (*c* 0.1, MeOH); UV (ACN) λ_max_: 244 nm; HRMS *m/z* 432.2901 ([M + H]^+^ calculated for C_29_H_38_NO_2_, *m/z* 432.2904; 0.8 ppm); ^1^H-NMR (DMSO-*d*_6_, 500 MHz): δ 0.77 (3H, d, 7.2, H11), 0.85 (3H, d, 6.7, H24), 1.13 (2H, m, H18), 1.23 (2H, m, H17), 1.38 (1H, m, H16), 1.41 (1H, m, H19), 1.56 (1H, m, H19'), 1.66 (3H, s, H12), 1.67 (1H, m, H15), 1.99 (1H, m,, H15'), 2.02 (1H, m, H20), 2.20 (1H, m, H5), 2.28 (1H, m, H20'), 2.40 (1H, dd, 13.2, 7.3, H10), 2.60 (1H, dd, 13.2, 4.9, H10'), 2.63 (1H, m, H8), 2.80 (1H, dd, 5.8, 2.6, H4), 3.25 (1H, m, H3), 5.11 (1H, ddd, 14.6, 10.3, 3.2, H14), 5.27 (1H, m, H7), 6.18 (1H, ddd, 15.2, 9.8, 1.7, H13), 6.54 (1H, ddd, 15.4, 10.2, 5.3, H21), 6.86 (1H, d, 15.5, H22), 7.10 (1H, d, 7.5, H26), 7.10 (1H, d, 7.5, H30), 7.16 (1H, d, 7.5, H28), 7.25 (1H, dd, 7.4, 1.0, H27), 7.25 (1H, dd, 7.4, 1.0, H29), 7.94 (1H, s, H2). ^13^C-NMR (DMSO-*d*_6_, 125 MHz) δ 12.6 (C11), 19.3 (C12), 20.8 (C24), 23.1 (C18), 25.2 (C19), 28.5 (C17), 31.1 (C20), 31.9 (C16), 33.7 (C5), 39.7 (C15), 43.1 (C10), 47.0 (C8), 47.2 (C4), 53.0 (C3), 65.7 (C9), 125.8 (C7), 126.0 (C28), 127.3 (C22), 127.9 (C27), 127.9 (C29), 129.3 (C13), 129.5 (C26), 129.5 (C30), 131.6 (C14), 136.7 (C25), 139.1 (C6), 145.7 (C21), 173.6 (C1), 196.9 (C23).

### 3.3. Chemical Analysis

Analysis of extracts was performed using ultra-high-performance liquid chromatography (UHPLC) UV/Vis diode array detector (DAD) high-resolution MS on a maXis 3G orthogonal acceleration quadrupole time of flight mass spectrometer (Bruker Daltonics, Bremen, Germany) equipped with an electrospray ionization (ESI) source and connected to an Ultimate 3000 UHPLC system (Dionex, Sunnyvale, CA, USA). The mass spectrometer was calibrated using sodium formate automatically infused prior to each analytical run, providing a mass accuracy below 1 ppm. Separation was achieved on a Kinetex C_18_, 2.6 µm, 2.1 × 100 mm column (Phenomenex) with a flow rate of 0.4 mL∙min^−1^ at 40 °C using a linear gradient 10% ACN in milliQ water going to 100% ACN in 10 min. Both solvents were buffered with 20 mM formic acid.

### 3.4. NMR and Optical Roation

One-dimensional and two-dimensional NMR experiments were acquired on a 500 MHz Varian Unity Inova (Palo Alto, CA, USA) equipped with a HCP probe. 1H, DQF-COSY, edHSQC, HMBC and NOESY experiments were acquired using standard pulse sequences. Optical rotation values were obtained on a Perkin-Elmer 241 Polarimeter at 589 nm.

### 3.5. CLL Cells, Cell Viability and Apoptosis Assays

Whole blood samples were obtained from healthy donors or patients that matched the standard diagnostic criteria for CLL after informed consent in accordance with the Declaration of Helsinki. All studies performed were approved by the ethics committee of the University of Ulm. Peripheral blood B cells were isolated by Ficoll density gradient followed by magnetic cell enrichment using CD19-MACS beads (Miltenyi Biotech, Bergisch Gladbach, Germany). Healthy donor B cells or CLL cells were cultured in conditioned medium of HS-5 cells, which was harvested after 3–4 days of culture and 80% confluency and depleted of HS-5 cells and debris by centrifugation. Cells were seeded in duplicates at a density of 3 × 10^5^ cells/well in opaque-walled 96-well plates. Pure compounds were added in different concentrations and incubated for 24 h. A final concentration of 0.1% DMSO was used as negative control and 100 µM fludarabine as positive control. Cell viability was assessed using CellTiter-Glo^®^ assay (Promega, Madison, WI, USA) according to manufacturer’s protocol. Luminescence signals were recorded using a Mithras LB940 plate reader (Berthold Technologies, Bad Wildbad, Germany). Relative cell viability was calculated as described by Knudsen *et al.* [10]. All compounds were tested on the same patient cells under the same conditions. For patient data, see Supplementary Table S2, Figures S17, S18.

## 4. Conclusions

In summary the two new cytochalasins, sclerotionigrin A (**1**) and B (**2**) have been isolated from *A*. *sclerotioniger*, together with the known proxiphomin (**3**). Compound **3** displayed the strongest cytotoxic effects towards CLL, however not as promising as recently demonstrated for chaetoglobosin A [10]. This is the first report of cytochalasan production from one of the currently more than twenty-five known black *Aspergillus* species [36,37], even though cytochalasans are very common in the related yellow *Aspergillus* species such as *A. flavipes*, *A. terreus* and *A. elegans* [29,38]. Further species in *Aspergillus* section *Nigri* should be examined for cytochalasan production, as the cytochalasan related metabolite, aspergillin PZ, has also been found in this group in addition to proxiphomin, and sclerotionigrin A and B. This may be important for both drug discovery and food safety, as the black Aspergilli are common in foods.

## Supplementary Materials

^1^H, DQF-COSY, HSQC, HMBC and NOESY spectra for all three compounds, as well as bioassay results are available in the supporting information. Supplementary materials can be accessed at: http://www.mdpi.com/1420-3049/19/7/9786/s1.
