# Supplementary material for: Isolation, Structural Analyses and Biological Activity Assays against Chronic Lymphocytic Leukemia of Two Novel Cytochalasins — Sclerotionigrin A and B
Source: Molecules. 2014 Jul 8;19(7):9786–97. doi: 10.3390/molecules19079786 (PMC6271702; doi:10.3390/molecules19079786)

Supplementary Material

Figure S1. ^{1}H-NMR spectrum for Sclerotionigrin A (1) at 500 MHz in DMSO-d_6.

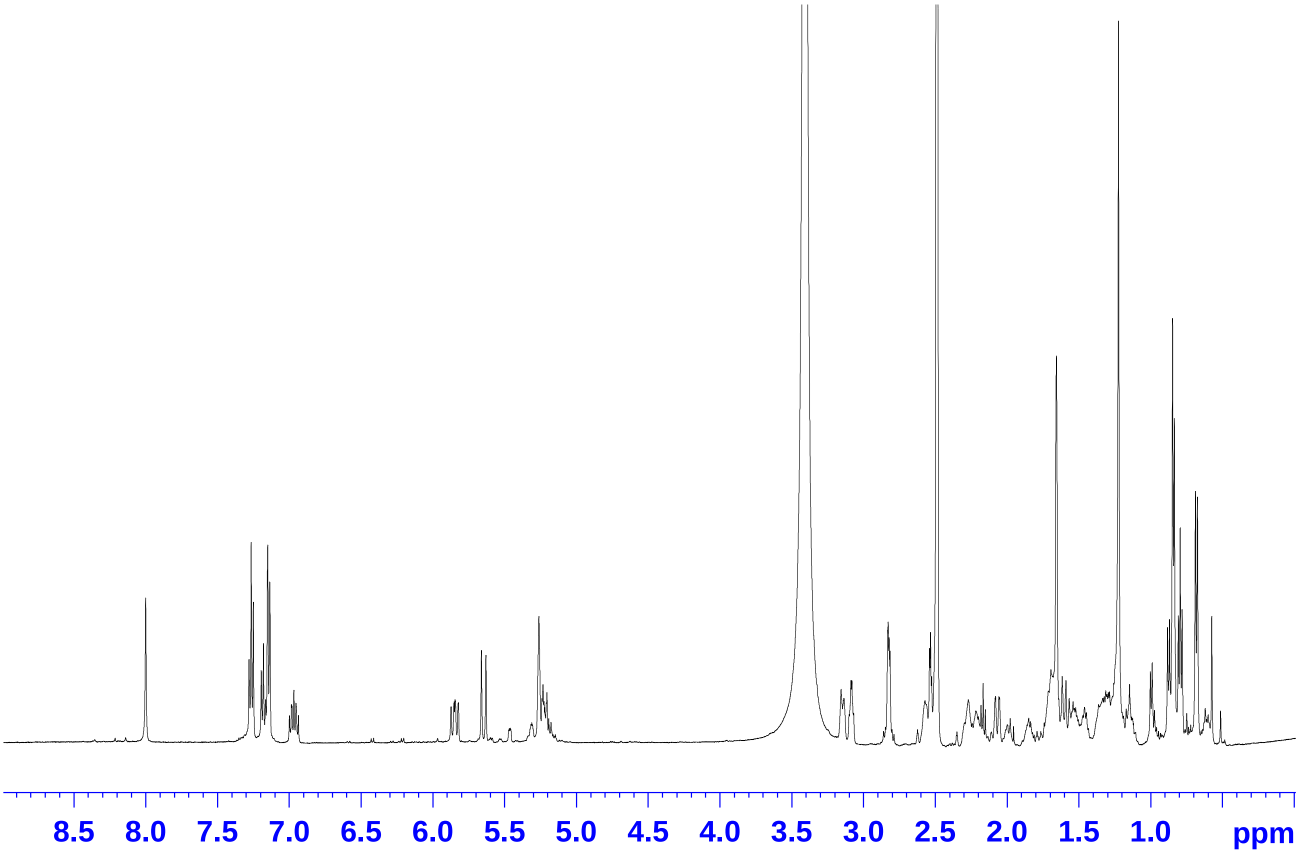

Figure S2. DQF-COSY spectrum for Sclerotionigrin A (1) in DMSO-d_6.

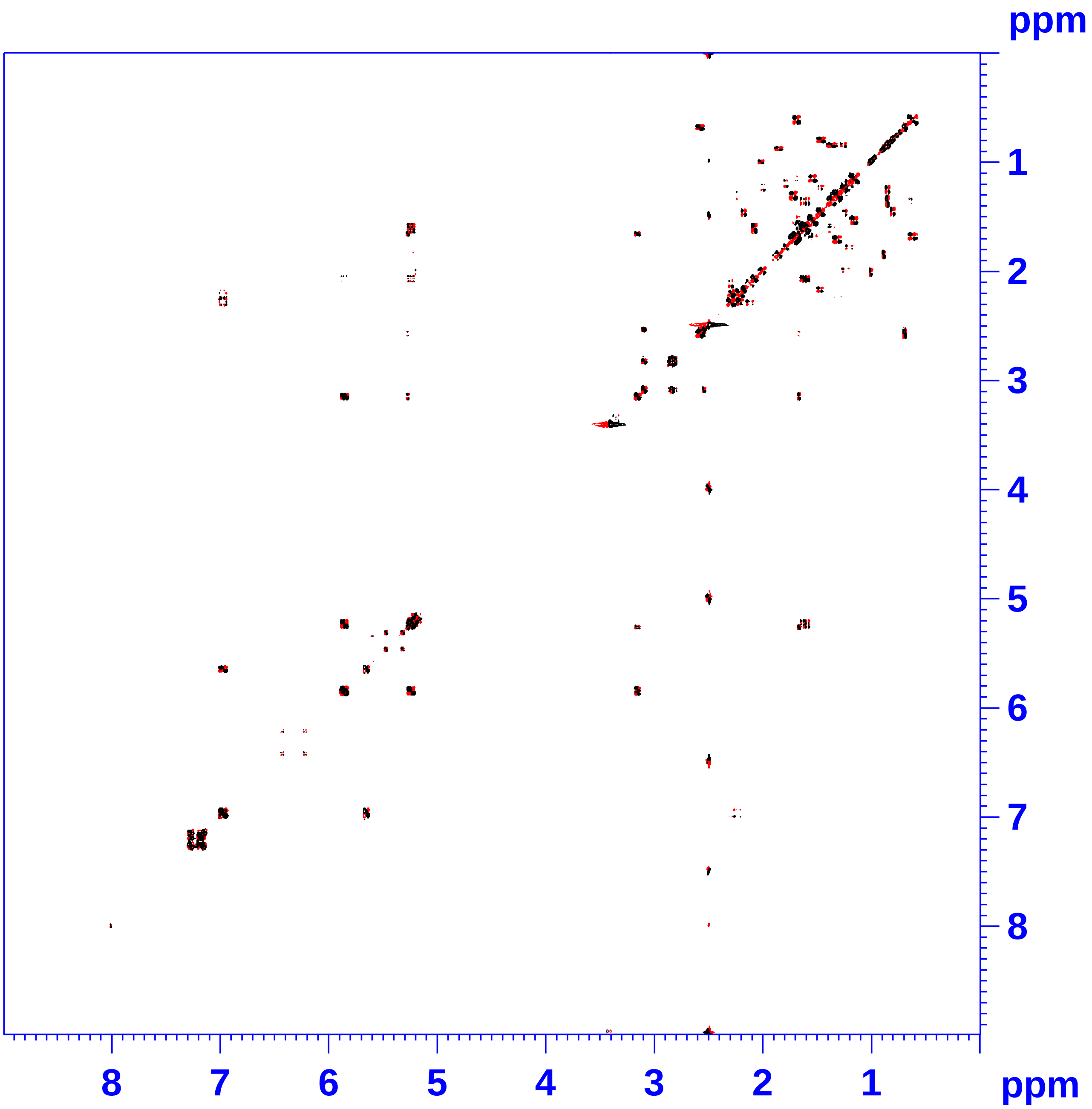

Figure S3. edHSQC spectrum for Sclerotionigrin A (1) in DMSO-d_6

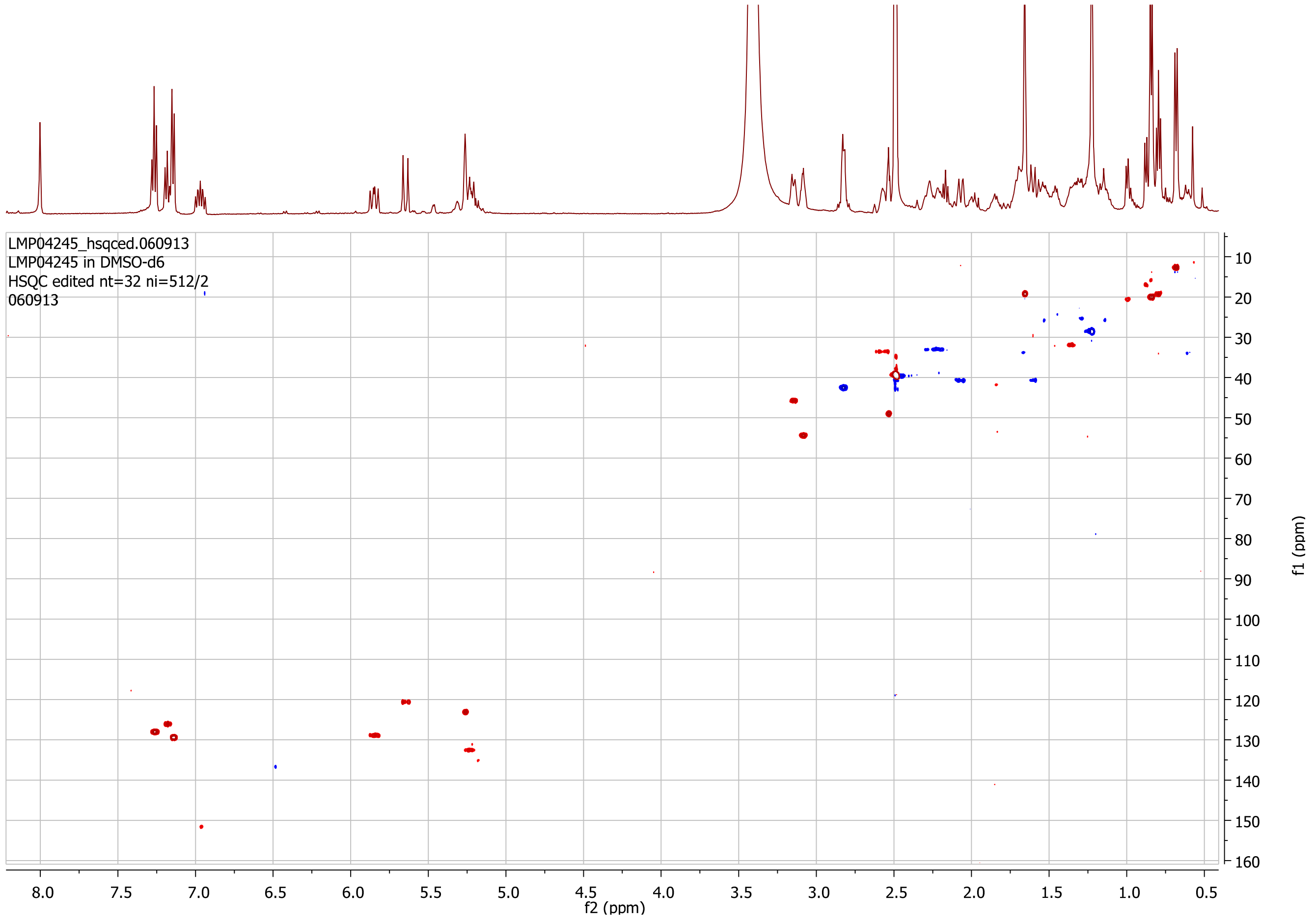

Figure S4. HMBC spectrum for Sclerotionigrin A (1) in DMSO-d_6.

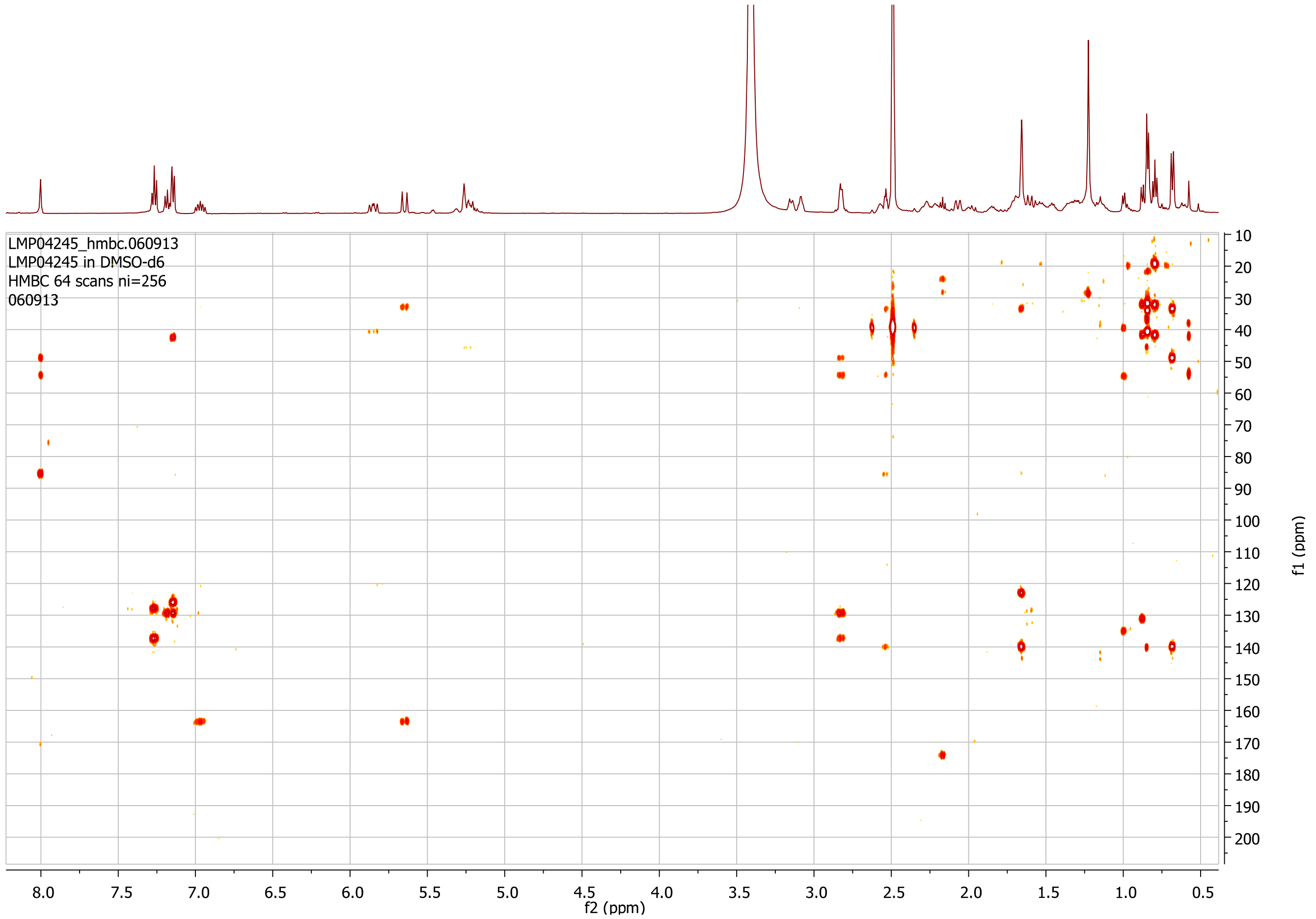

Figure S5. NOESY spectrum for Sclerotionigrin A (1) in DMSO-d_6

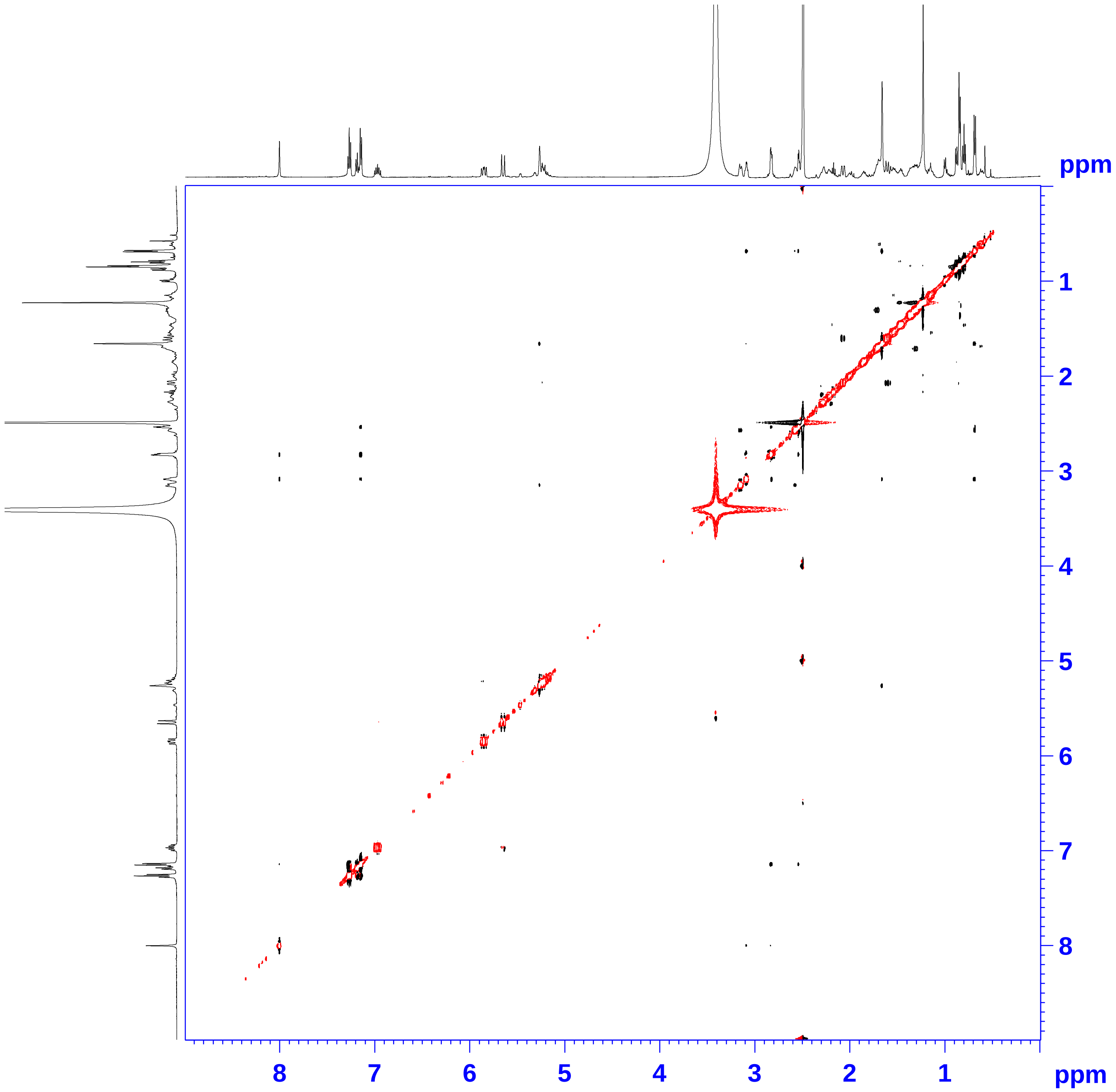

Figure S6. ^{1}H-NMR spectrum for Sclerotionigrin B (2) at 500 MHz in DMSO-d_6.

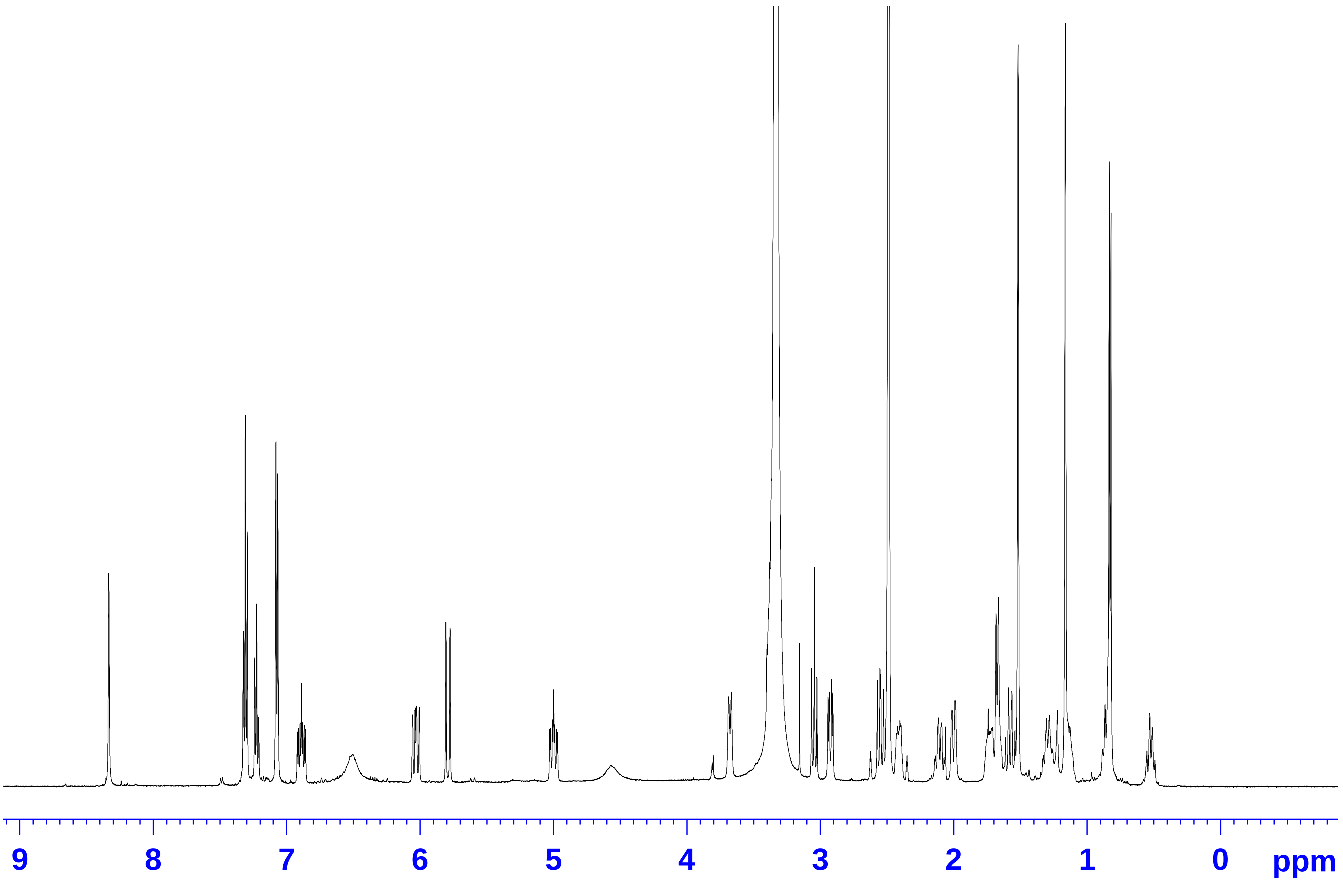

Figure S7. DQF-COSY spectrum for sclerotionigrin B (2) in DMSO-d_6.

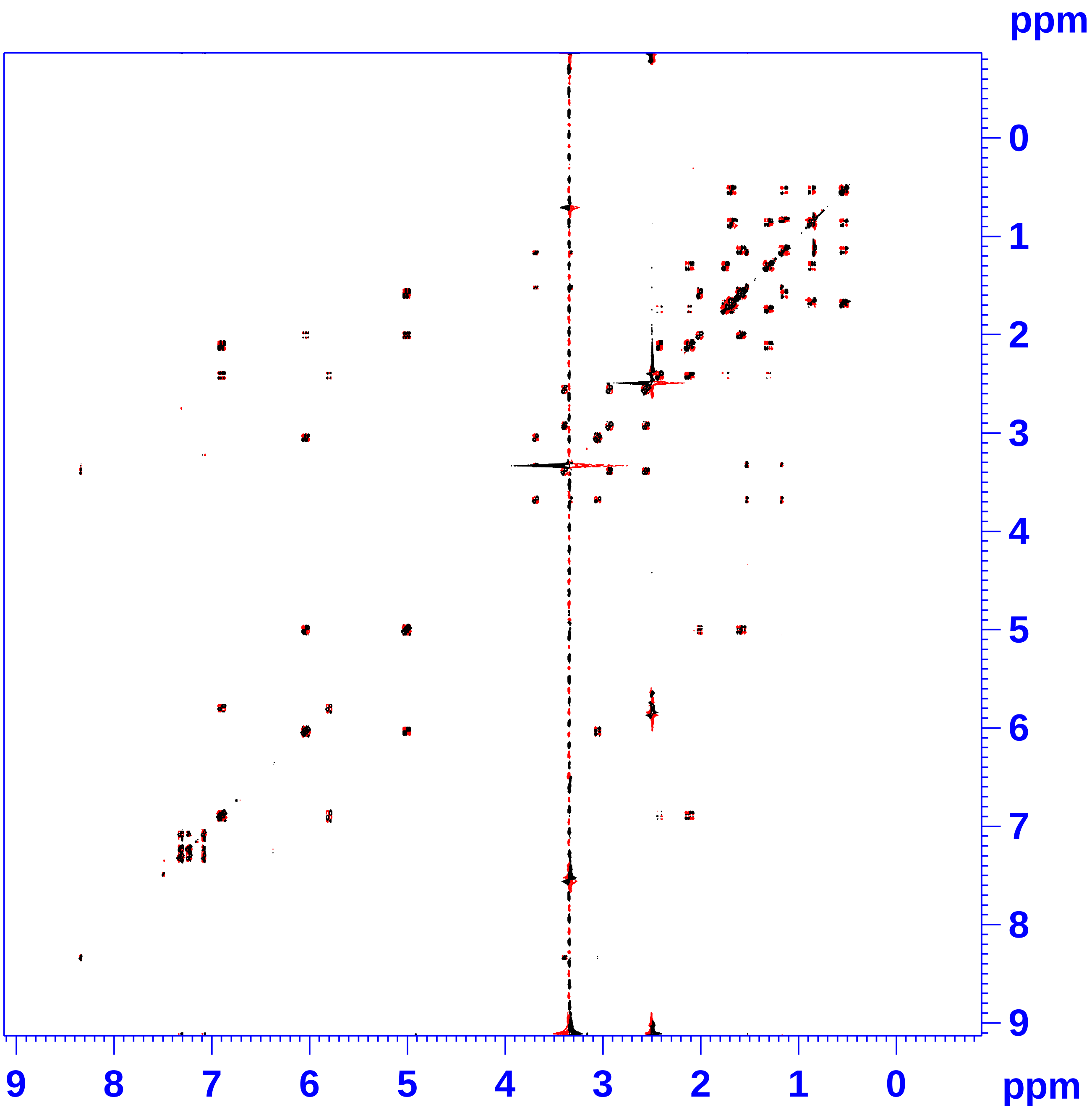

Figure S8. $_{ed}$HSQC spectrum for Sclerotionigrin B (2) in DMSO-d_6.

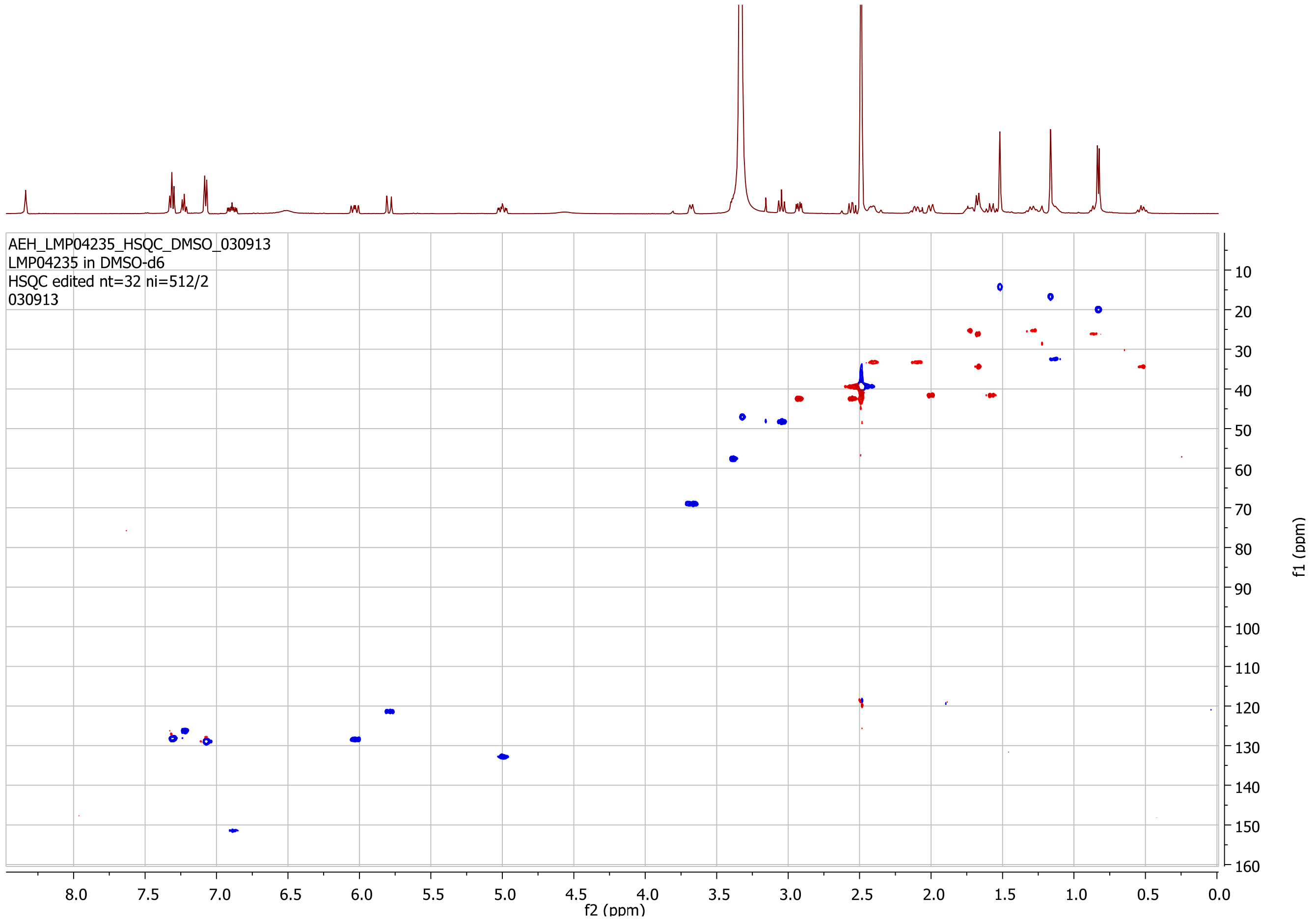

Figure S9. HMBC spectrum for Sclerotionigrin B (2) in DMSO-d_6.

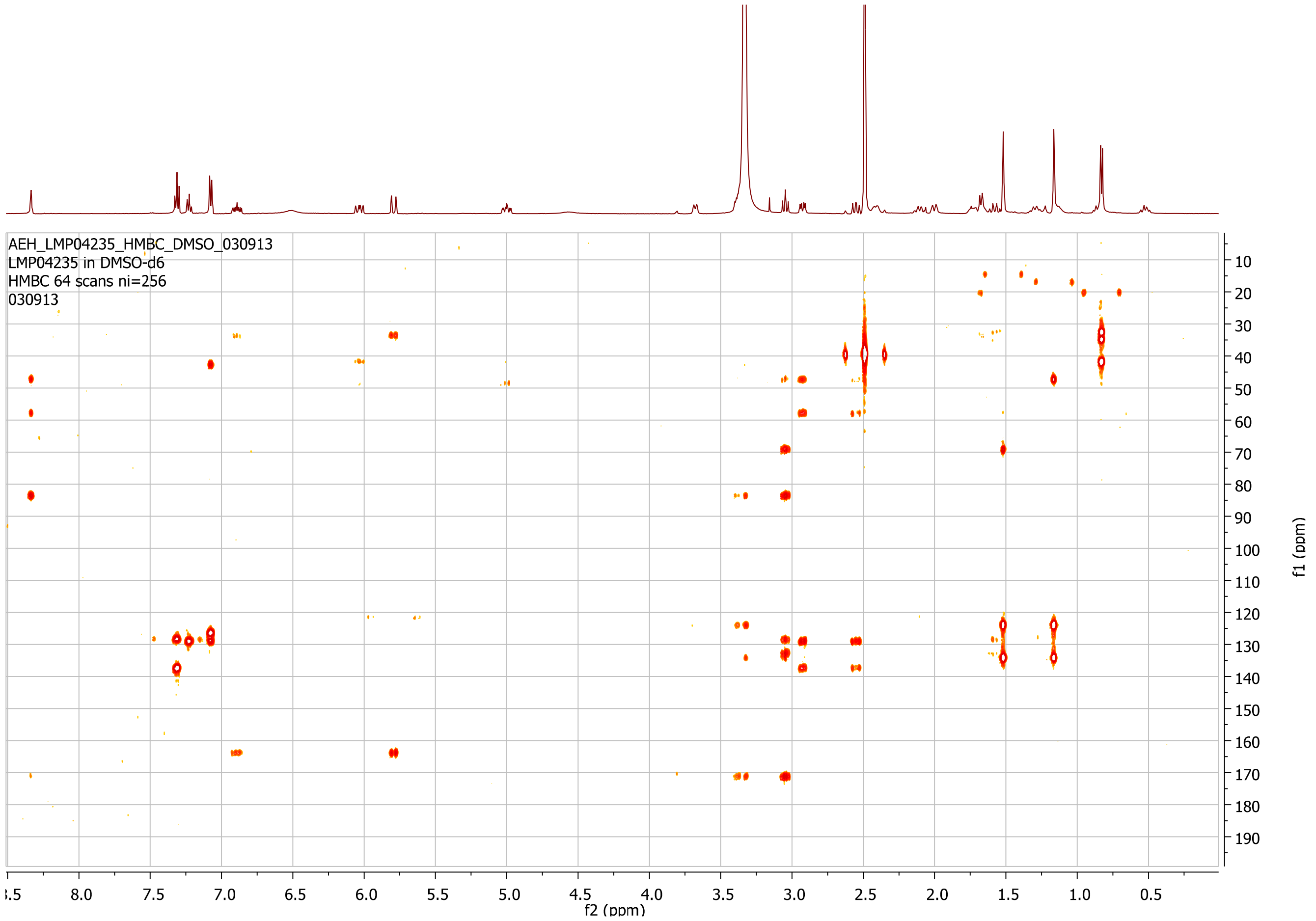

Figure S10. NOESY spectrum for Sclerotionigrin B (2) in DMSO-d_6.

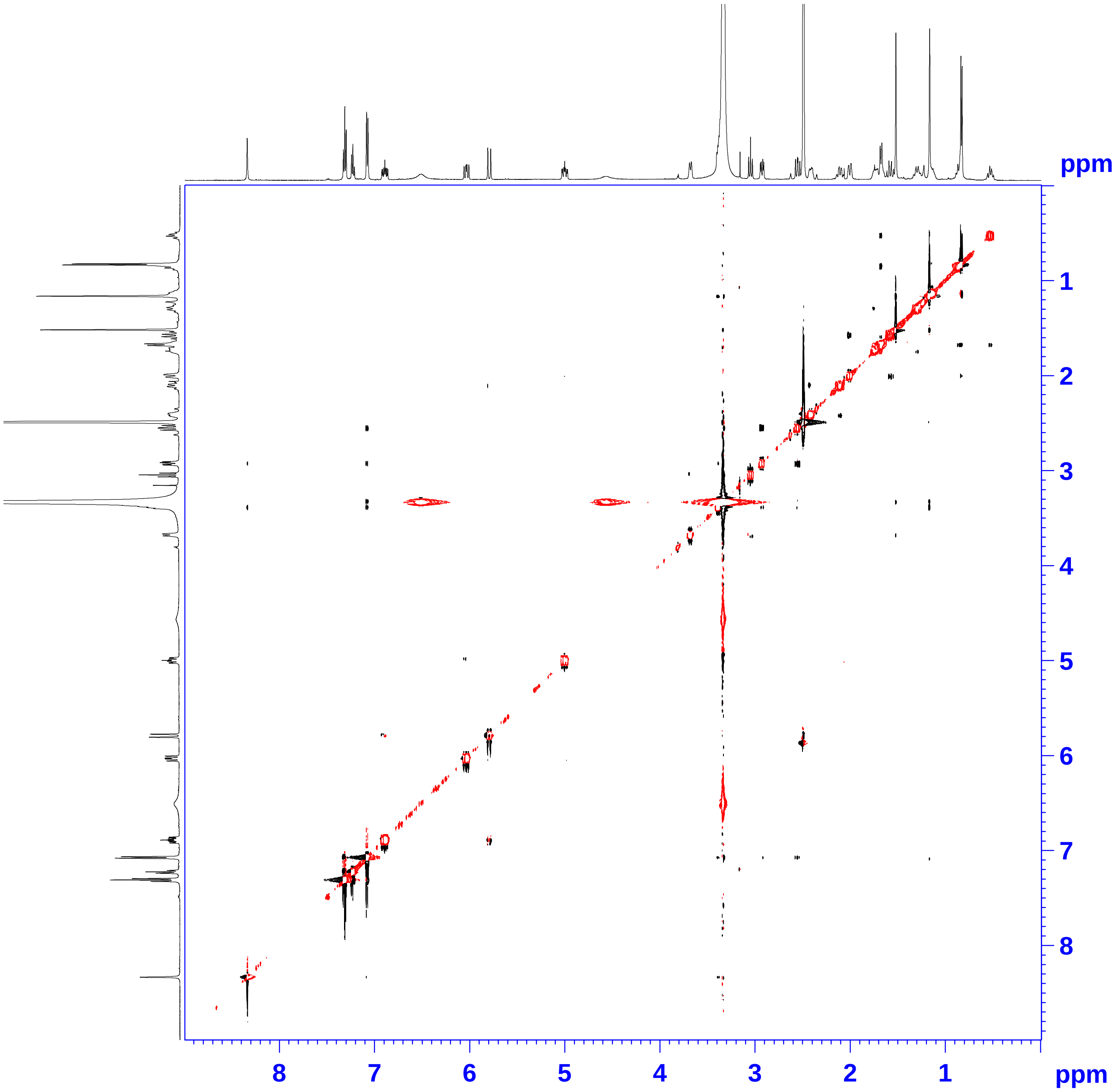

Figure S11. NMR data for proxiphomin (3).

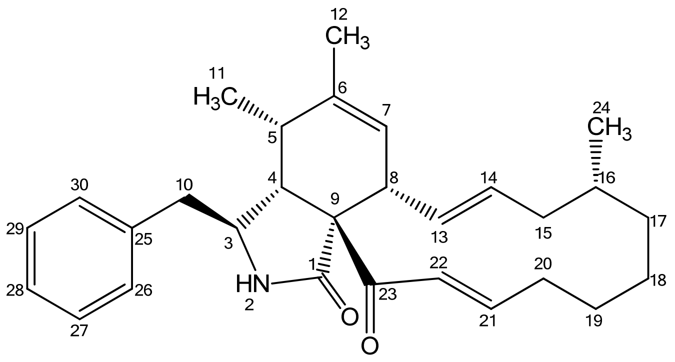

Table S1. NMR data for proxiphomin (3).

No.	δ_H (integral, mult., J [Hz])†	^{13}C-chemical shift [ppm]†	HMBC correlations	NOESY connectivities
1	-	173.6	-	-
2	7.94 (1H, s)	-	3, 4, 9	3
3	3.25 (1H, m)	53.0	-	2, 10, 10', 11, 12, 26, 30
4	2.80 (1H, dd, 5.8, 2.6)	47.2	1, 3, 5, 6, 9, 10, 23	5, 10, 10', 11, 26, 30
5	2.20 (1H, m)	33.7	1	4, 8, 11
6	-	139.1	-	-
7	5.27 (1H, m)	125.8	-	8, 12, 13
8	2.63 (1H, m)	47.0	1	7, 5, 13, 14, 22
9	-	65.7	-	-
10	2.40 (1H, dd, 13.2, 7.3)	43.1	1, 3, 4, 25, 26, 30	3, 4, 10', 26, 30
10'	2.60 (1H, dd, 13.2, 4.9)	43.1	1, 3, 4, 25, 26, 30	3, 4, 10, 26, 30
11	0.77 (3H, d, 7.2)	12.6	4, 5, 6	3, 4, 5
12	1.66 (3H, s)	19.3	5, 7	3, 7, 13
13	6.18 (1H, ddd, 15.2, 9.8, 1.7)	129.3	15	7, 8, 12, 14, 22
14	5.11 (1H, ddd, 14.6, 10.3, 3.2)	131.6	8	8, 13, 15'
15	1.67 (1H, m)	39.7	16	15'
15'	1.99 (1H, m)	39.7	16	14, 15, 16, 17 24
16	1.38 (1H, m)	31.9	-	15'
17	1.23 (2H, m)	28.5	-	15', 18
18	1.13 (2H, m)	23.1	-	17
19	1.41 (1H, m)	25.2	-	19'
19'	1.56 (1H, m)	25.2	-	19
20	2.02 (1H, m)	31.1	-	20', 21, 22
20'	2.28 (1H, m)	31.1	-	20
21	6.54 (1H, ddd, 15.4, 10.2, 5.3)	145.7	20, 23	20
22	6.86 (1H, d, 15.5)	127.3	20, 23	8, 13, 20
23	-	196.9	-	-
24	0.85 (3H, d, 6.7)	20.8	15, 16	15'
25	-	136.7	-	-
26‡	7.10 (1H, d, 7.5)	129.5	10, 28, 30	3, 4, 10, 10'
27‡	7.25 (1H, dd, 7.4, 1.0)	127.9	25, 29	
28	7.16 (1H, d, 7.5)	126.0	26, 30	
29‡	7.25 (1H, dd, 7.4, 1.0)	127.9	25, 27	
30‡	7.10 (1H, d, 7.5)	129.5	10, 26, 28	3, 4, 10, 10'

† ^{1}H-NMR data were obtained at 500 MHz in DMSO-d_6 and ^{13}C data were obtained at 125 MHz in DMSO-d_6.
‡It was not possible to distinguish between 26 and 30 as well as 27 and 29.

Figure S12. ^{1}H-NMR spectrum for proxiphomin (3) at 500 MHz in DMSO-d_6.

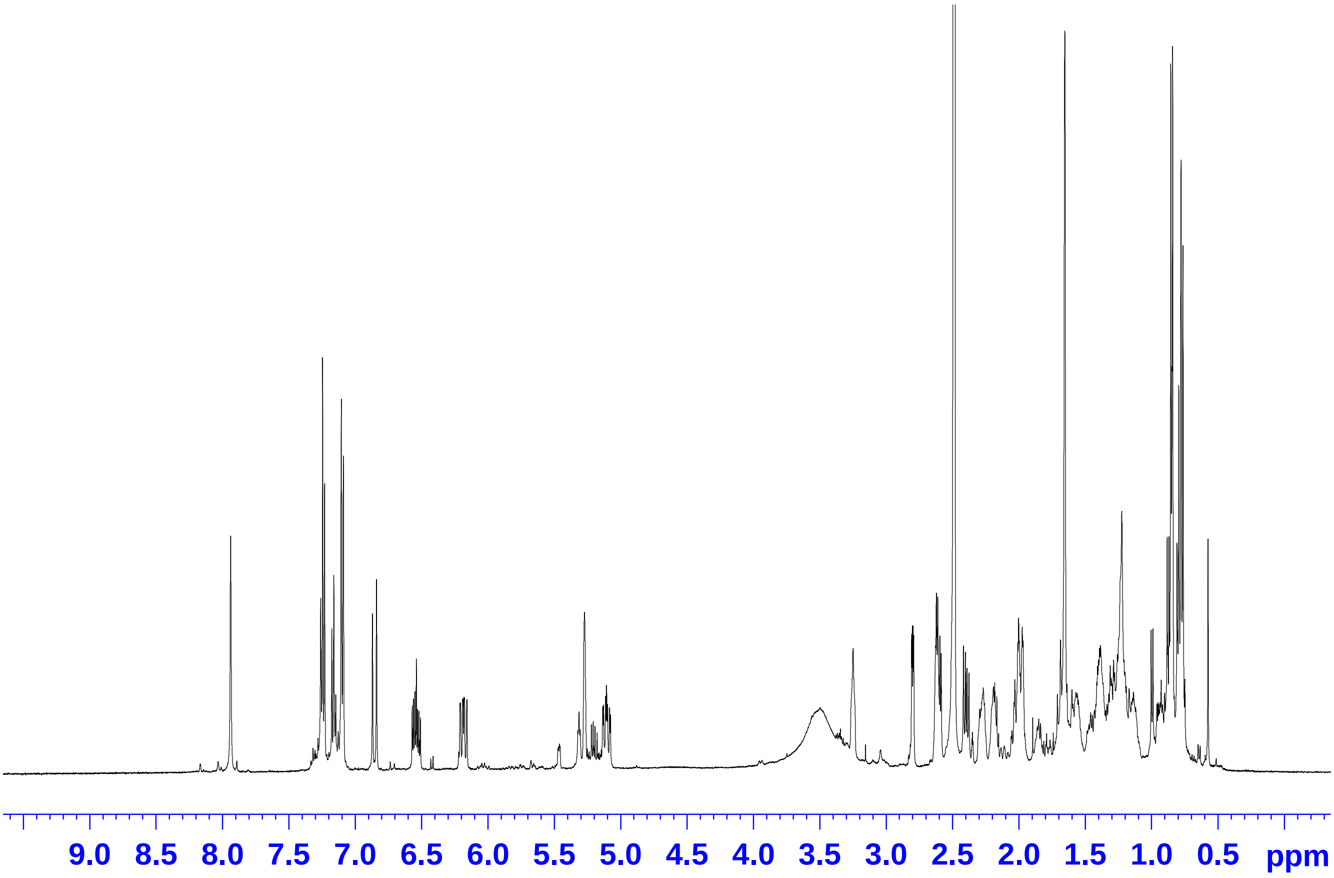

Figure S13. DQF-COSY spectrum for proxiphomin (3) in DMSO-d_6.

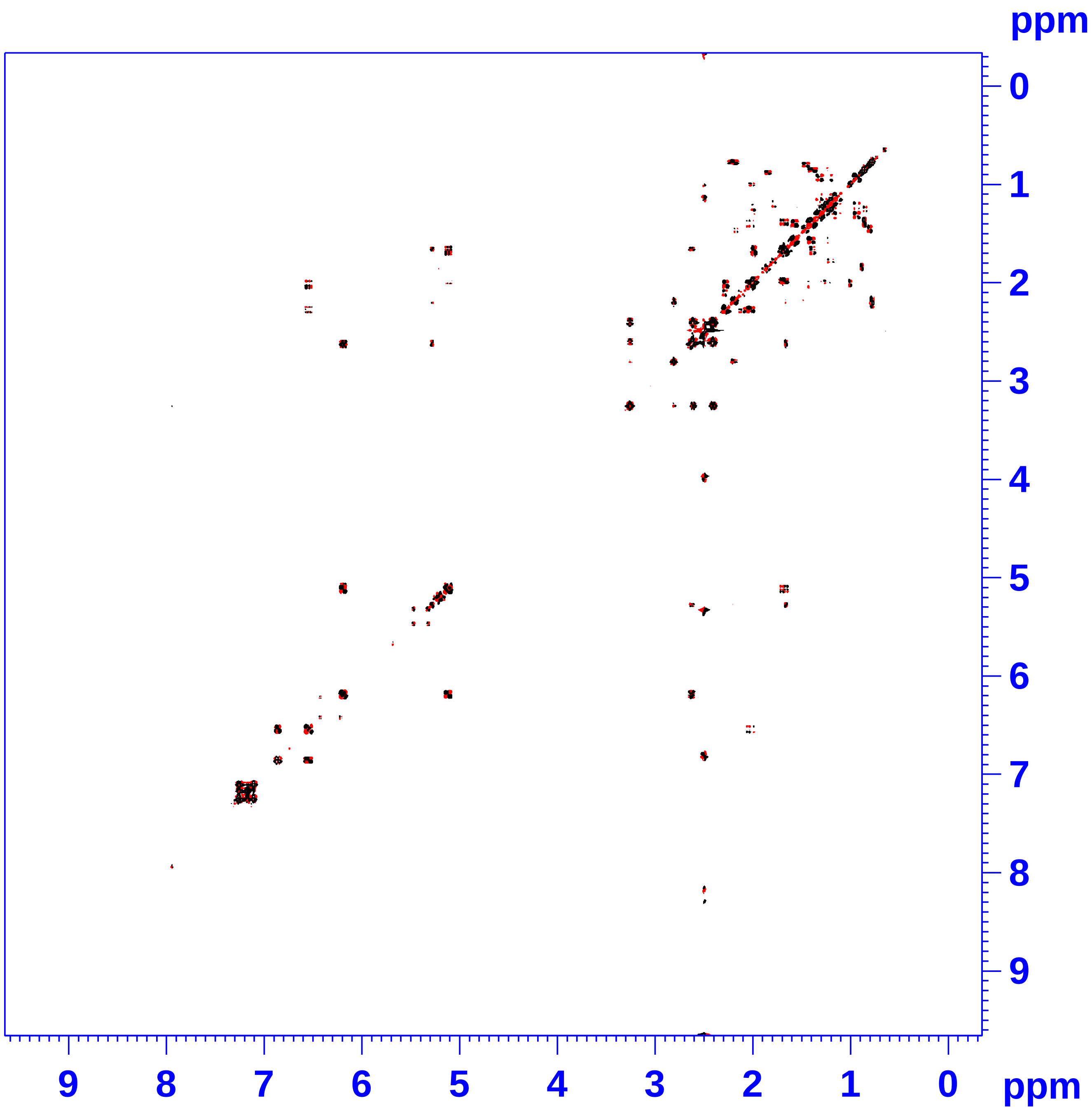

Figure S14. $_{ed}$HSQC spectrum for proxiphomin (3) in DMSO-d_6.

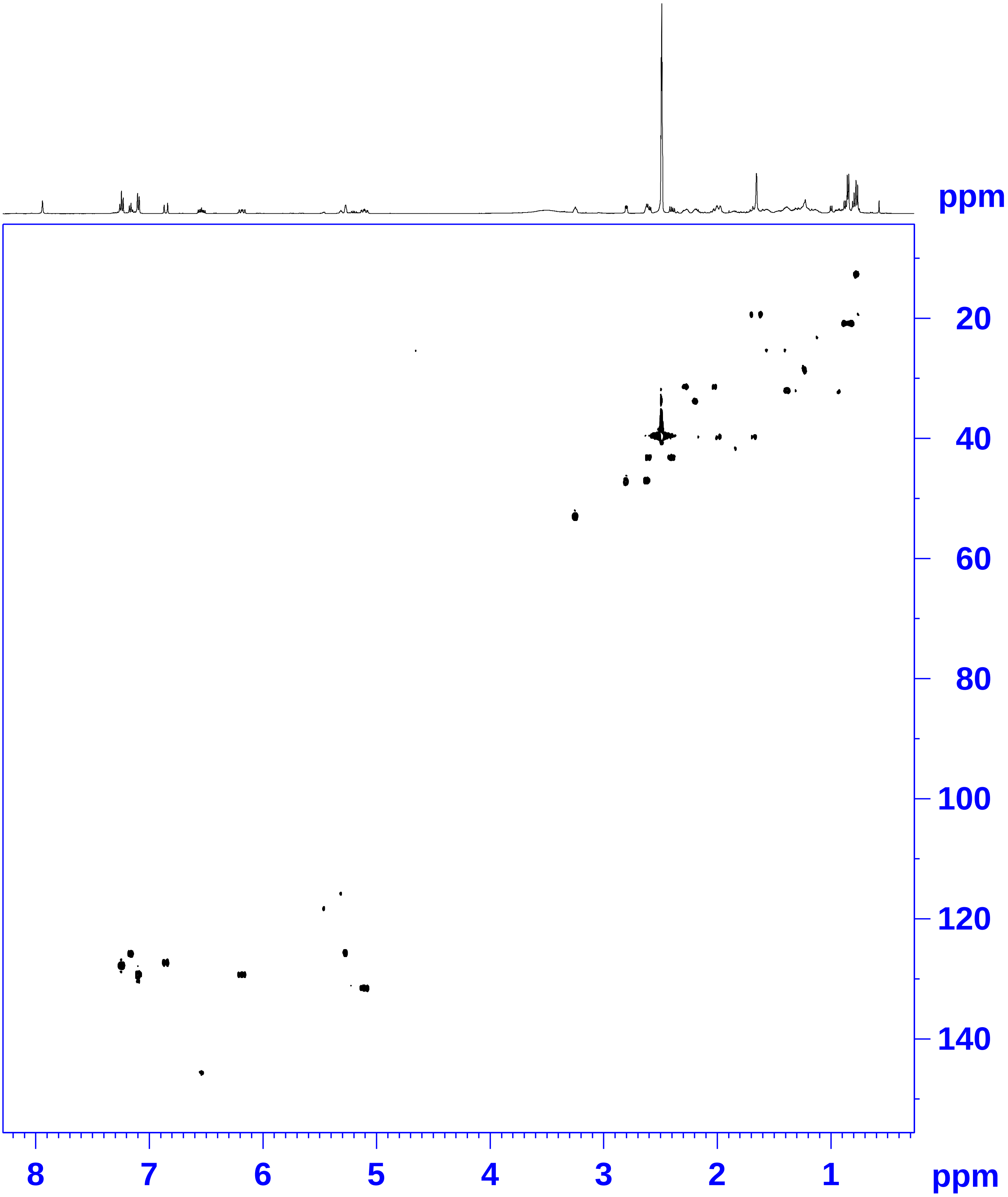

Figure S15. HMBC spectrum for proxiphomin (3) in DMSO-d_6

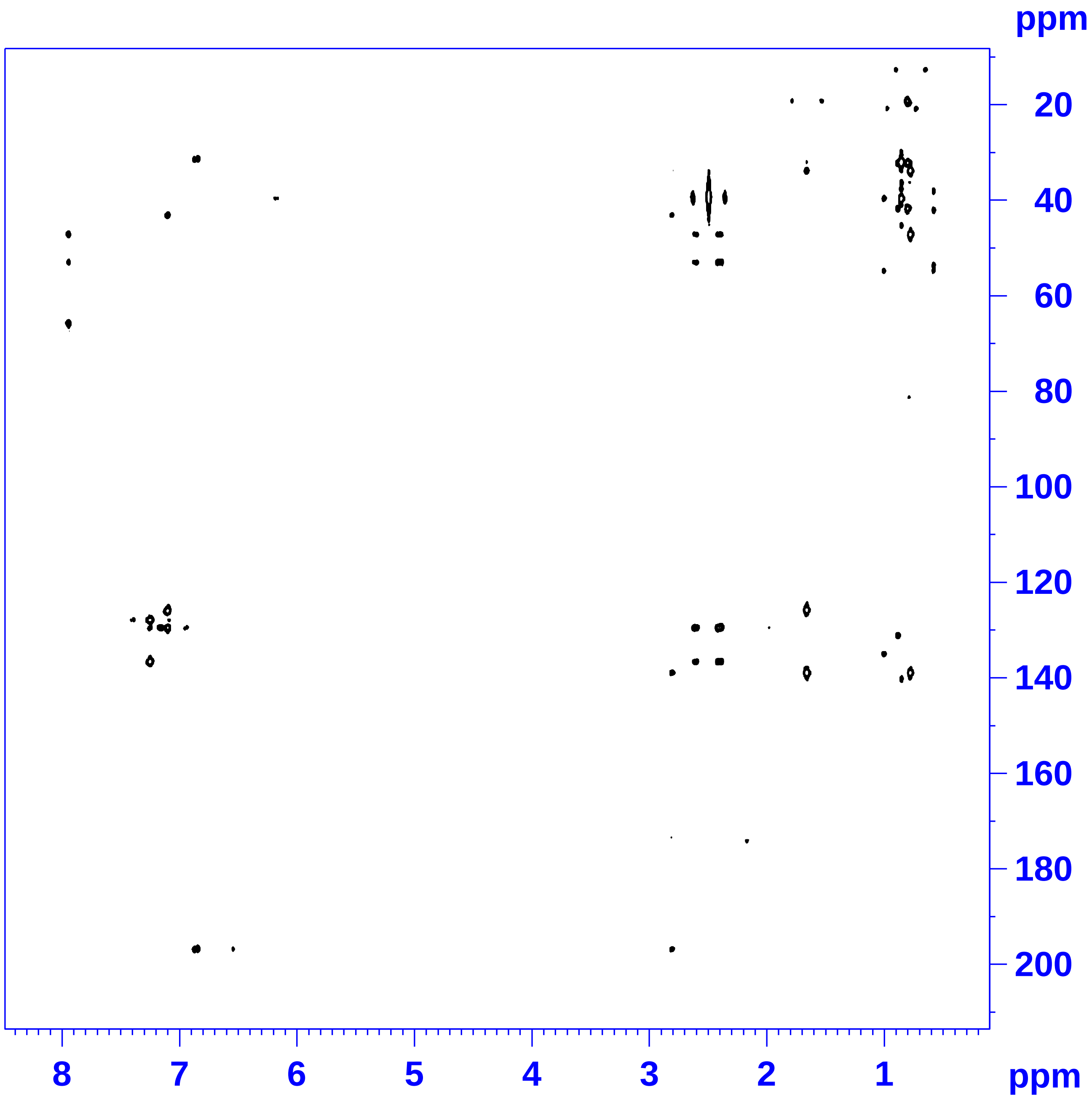

Figure S16. NOESY spectrum for proxiphomin (3) in DMSO-d_6.

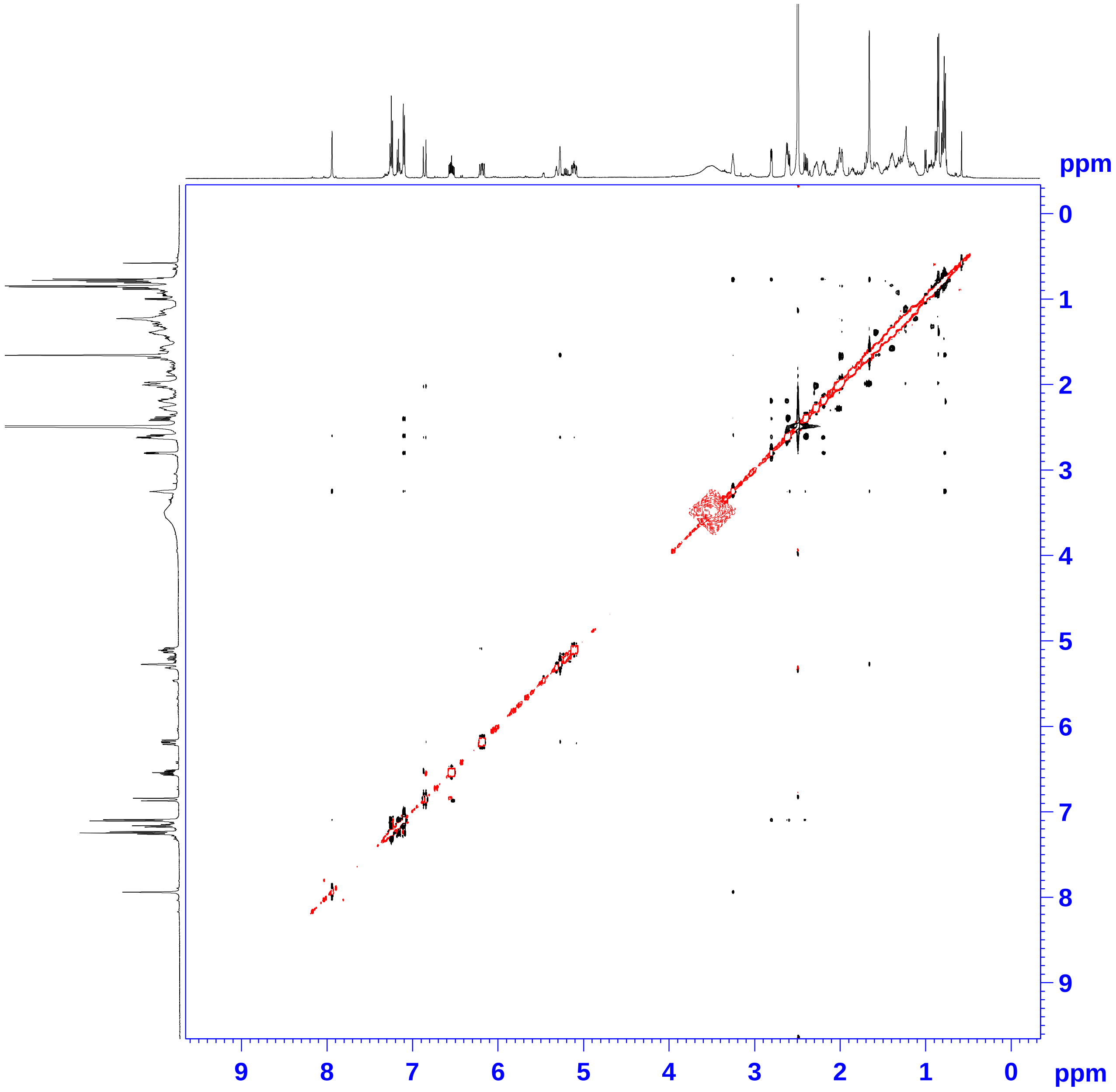

Table S2. Patient data, including age, clinical stage, IGHV mutational status and fluorescence in situ hybridization (FISH) results.

Number	Age	Binet	IGHV	FISH
**1**	55	A	unmutated	13q-
**2**	64	A	mutated	13q-
**3**	48	A	mutated	13q-, Tris 18
**4**	53	A	mutated	13q-

Mean values ± SEM of four CLL samples and three healthy donor samples are depicted.

Figure S17. Effects of 1–3 on CLL cell viabiltiy. CLL cells were treated for 24 h with increasing concentrations of 1–3 and cell viability was analyzed by CellTiter-Glo® assay. Relative cell viability is compared to DMSO control (0.1%).

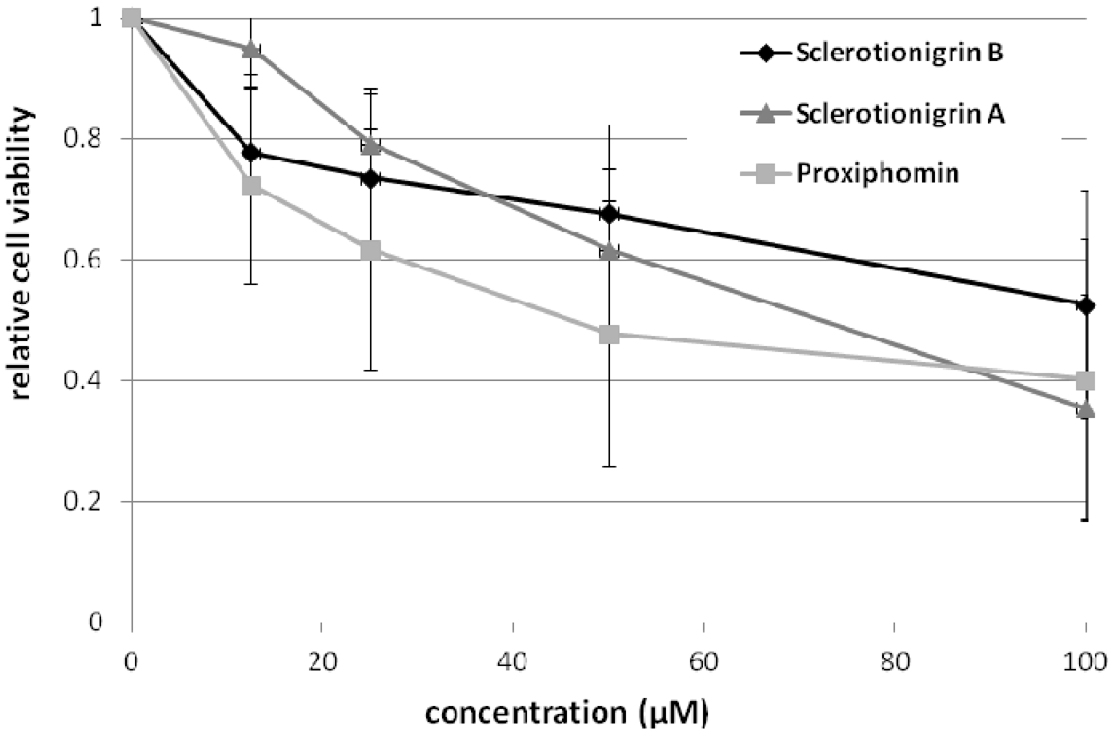

Figure S18. Effects of 1–3 on healthy B-cells cell viabiltiy. Healthy B-cells were treated for 24 h with increasing concentrations of 1–3 and cell viability was analyzed by CellTiter-Glo® assay. Relative cell viability is compared to DMSO control (0.1%).

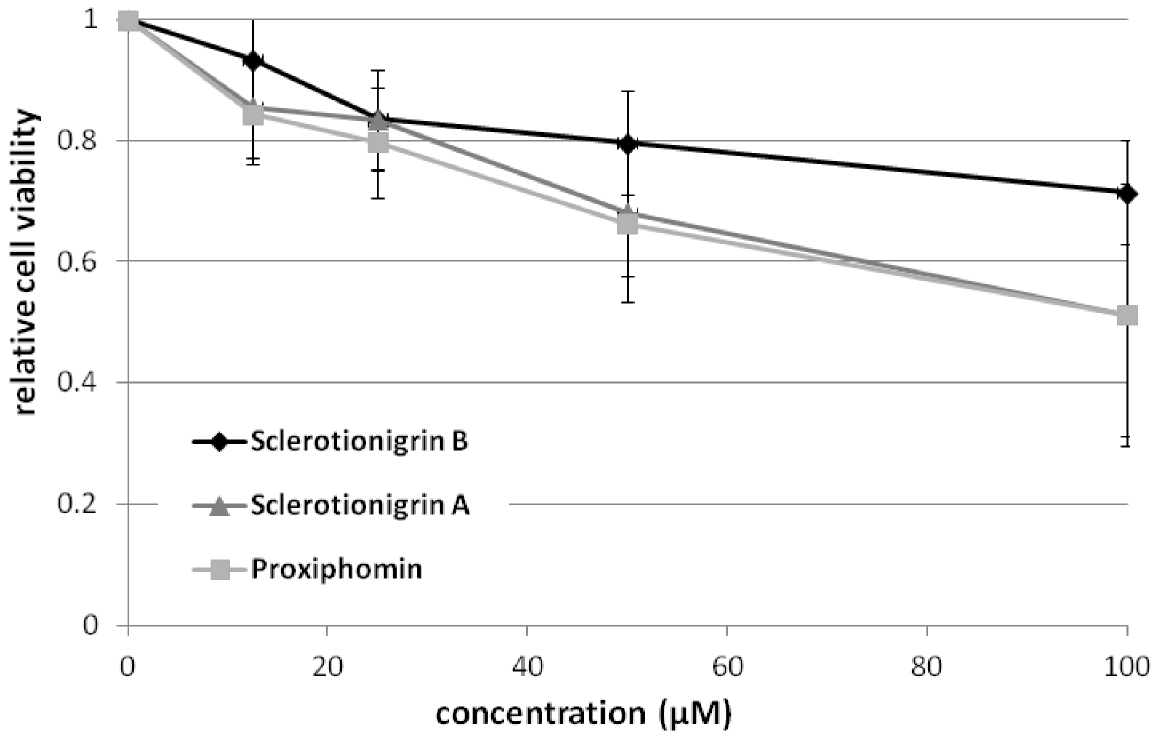

Supplement: Supplementary file 1 [file molecules-19-09786-s001.pdf]
